# Surface Activity of Hydrophobized Modified Starch Hydrolysates in Mixed Systems

**DOI:** 10.3390/ma17225526

**Published:** 2024-11-12

**Authors:** Emilia Konował, Marta Sybis, Krystyna Prochaska

**Affiliations:** 1 Institute of Chemistry and Technical Electrochemistry, Poznan University of Technology, Berdychowo 4, 60-965 Poznan, Poland; emilia.konowal@put.poznan.pl; 2 Department of Construction and Geoengineering, Poznan University of Life Sciences, Piatkowska 94 E, 60-649 Poznan, Poland; marta.sybis@up.poznan.pl; 3 Institute of Chemical Technology and Engineering, Poznan University of Technology, Berdychowo 4, 60-965 Poznan, Poland

**Keywords:** chemically modified starch, dynamic surface tension, emulsion stability, polysaccharide emulsifiers, surface activity, surface mole fraction, synergistic effect

## Abstract

The manuscript presents research focusing on the adsorption and emulsion properties of starch hydrolysates modified through acetylation, oxidation, and cross-linking. The techniques used in this study included measurements of equilibrium surface tension (du Noüy ring) dynamic surface tension (drop shape analysis), and the preparation and evaluation of emulsion stability (TURBISCAN). The surface activity of the acetylated starch hydrolysates is affected by the degree of acetylation. The acetylated starch 0.02Ac-H exhibited higher surface activity than the more highly substituted derivative 0.1Ac-H. Furthermore, it was shown that the surface activity of the components increased as the acetylated oxidized starch underwent hydrolysis. The fractions collected after 180 min using a membrane with a low separation capability (8 kDa) revealed the highest capacity for reducing surface tension. In binary systems consisting of starch derivatives and surfactants, synergistic effects in reducing surface tension were particularly noticeable in systems containing ionic surfactants. The addition of a cationic surfactant to the modified starch hydrolysate solution (1:6 mol/mol) resulted in a significantly more efficient saturation of the air/water interface. This study demonstrated that emulsions stabilized with modified starch hydrolysates remained stable over time, even when these hydrolysates constituted up to 60% of the emulsifier mixture.

## 1. Introduction

The growing interest in saccharide surfactants observed in recent years is primarily driven by environmental considerations, as these compounds are produced almost exclusively from renewable resources, exhibit no toxicity, and are fully biodegradable. Moreover, they possess favorable surface properties, which facilitate their complete or partial replacement of synthetic substances in various industries, including food, cosmetics, agriculture, pharmaceuticals, and detergent manufacturing [1,2].

Hydrophobized biopolymers can adopt two types of conformations at the air–solution interface:

- The hydrophobic groups are oriented towards the air phase, while the hydrophilic groups are fully immersed in the aqueous phase;
- The hydrophobic groups are embedded or anchored within micellar aggregates, rendering them inaccessible for polymer adsorption at the interfacial surface.

Studies on the surface activity of hydrophobically modified biopolymers confirm their high surface activity at both air/water and oil/water phase interface.

The literature provides numerous reports on the surface activity of sugars and hydrophobically modified saccharide biopolymers [3,4,5,6,7,8,9,10,11,12], including glucose, sucrose, raffinose, and stachyose esters [5]; hydrophobized dextran T40 derivatives [13]; and carboxymethylpullulan derivatives [6]; as well as lactose, fructose, and maltodextrin [12,14].

For example, R. Shogren and G. Biresaw [11] conducted studies on the surface activity of aqueous solutions of maltodextrins and starch esters. The authors demonstrated that both maltodextrin and the starch esters studied exhibited surface activity in the air/water and hexadecane–water systems. In both systems, an increase in surface activity was observed with increasing biopolymer concentration in the solution. The authors determined the critical values of surface and interfacial tension for starch acetate to be 41–44 mN/m and 11–14 mN/m, respectively. In the case of maltodextrin, these values were 56 and 22 mN/m, respectively. These results were obtained for solutions with concentrations above 20%.

Additionally, the cited authors investigated the adsorption properties of 1% aqueous solutions of starch modified with octenyl succinic anhydride (OSA) and dodecyl succinic anhydride (DDSA). They showed that even a small degree of substitution (DS), with derivatives having DS values of 0.02–0.04, caused a measurable reduction in surface tension at the air/water interface to values of 42–43 mN/m, and interfacial tension in the hexadecane–water system to values of 13–14 mN/m.

P. Erni and colleagues [7], L. Nilsson and B. Bergenstahl [9,10,15], and K. Prochaska et al. [14] investigated the adsorption properties of OSA-modified starch. These researchers demonstrated that sodium octenyl succinate starch is active at both the air/water and water/oil interfaces, which enhances its stabilizing and emulsifying properties during emulsion formation.

Studies on the adsorption capacity in air/water and toluene–water systems for both native starch and various chemically modified potato starch preparations were conducted by K. Prochaska, G. Lewandowicz, and colleagues [16]. The authors analyzed several commercial biopolymers, such as oxidized starch, distarch phosphate, acetylated starches, acetylated distarch adipate, and acetylated distarch phosphate. Surface and interfacial tension measurements were performed using the Du Noüy ring method. It was found that the studied starch preparations exhibited higher surface activity compared to native starch.

Substances capable of reducing surface tension play a crucial role in the stability of emulsions. The emulsifier dissolves simultaneously in both phases and adsorbs at the interface between them, resulting in a higher concentration in the surface layer than in each phase [17]. An emulsifier can be an individual chemical compound or a mixture of substances. There are two main classes of emulsifiers: low-molecular-weight emulsifiers, such as monoglycerides, polysorbates, lecithin, and sugar esters, as well as high-molecular-weight emulsifiers, including proteins and polysaccharides [18,19].

A stabilizer ensures the long-term stability of an emulsion. These are typically biopolymers, such as proteins or polysaccharides [18,20,21]. A stabilizer functions primarily by altering the viscosity of the aqueous continuous phase or by forming a stabilizing layer around oil droplets, as seen with proteins that exhibit a strong tendency to adsorb at the oil/water interface [19]. For a biopolymer to effectively act as an emulsifier or stabilizer, it must have amphiphilic properties, exhibit a strong tendency to adsorb, and be able to saturate the phase boundary. Additionally, it should be capable of forming a thick spatial or charged stabilizing layer [19,22].

The most commonly used polysaccharide emulsifiers in the food industry include gum arabic, modified starches, modified cellulose, and certain types of pectin and galactomannan. The surface activity of these hydrocolloids is attributed to their amphiphilic structure, i.e., the nonpolar nature of substituents attached to the hydrophilic backbone (as seen in hydrophobically modified starch or cellulose) or the presence of a protein component covalently or physically linked to the polysaccharide (as in some gums or pectins) [23,24,25,26].

An example of a modified starch with excellent emulsifying and stabilizing properties is hydrophobically modified sodium octenyl succinate starch. It exhibits high surface activity, similar to products of enzymatic hydrolysis of modified starches [15,27,28,29]. Modified starches and cellulose form a sticky adsorption layer at the liquid–liquid interface [23]. Another group of emulsifiers/stabilizers consists of high-molecular-weight glycoproteins, i.e., combinations of proteins and polysaccharides, which combine the properties of both proteins and polysaccharides and strongly adsorb at the interfacial surface [20].

Despite the presence of emulsifiers, an emulsion always remains a system with a tendency toward destabilization because it is impossible to reduce the interfacial tension between phases to zero, and thus prevent the system’s tendency to reduce entropy. For emulsions, the primary way to reduce entropy is by decreasing the phase contact area through the coalescence of dispersed phase droplets, which in turn reduces the interfacial tension [17].

Compounds synthesized from renewable resources are gaining increasing interest due to the benefits they offer for consumer health and environmental protection compared to products obtained through chemical synthesis. These compounds, being non-toxic and biodegradable, are ideal raw materials for use in cosmetic and food emulsion formulations, provided they exhibit surface-active properties. When they meet this requirement, they find applications in various fields, serving as thickeners and emulsion stabilizers.

Previous studies have clearly demonstrated that starch derivatives can effectively modify various properties of cement composites, thereby enhancing their functionality. The appropriate selection of additives and admixtures contributes to improved workability, water resistance, and frost resistance [30,31,32]. Regarding resistance to cyclic freezing and thawing, it is particularly essential to ensure adequate air entrainment in concrete, which involves introducing fine air bubbles into the mix to allow the freezing water to expand without damaging the concrete structure. Air entrainment in concrete is mainly achieved through the use of chemical admixtures containing surface-active agents, including anionic, amphoteric, and nonionic surfactants. The use of surfactants also improves the workability of concrete without altering the water/cement ratio, thereby optimizing the mechanical properties. Unfortunately, these surfactants are often synthetic compounds, which can have an adverse environmental impact. Therefore, an essential issue is the examination of the surface activity of binary systems such as modified starch hydrolysate–surfactant, which constitutes the aim of this publication. Such systems may reduce the proportion of synthetic surfactant in the admixture modifying the properties of cement composites in favor of a biopolymer derived from natural sources. Furthermore, in complement to the studies presented in the previous publication [33], this research also focuses on the production of emulsion systems incorporating a surface-active potato starch derivative.

## 2. Materials and Methods

In this study, the following modified starch hydrolysates were utilized:

- Acetylated starch with a degree of substitution of 0.02 (designated as 0.02Ac-H);
- Acetylated starch with a degree of substitution of 0.1 (0.1Ac-H);
- Cross-linked acetylated distarch phosphate (Ac-FD-H);
- Oxidized acetylated starch with an oxidation degree of 0.04 and an acetylation degree of 0.1 (Ac-Ox-H).

The starch preparations were kindly provided by the Institute of Agricultural and Food Biotechnology, Department of Food Concentrates and Starch Products, Poznań, Poland. The hydrolysates were obtained through enzymatic hydrolysis performed in an enzymatic membrane reactor with an external ultrafiltration module. A detailed procedure for the preparation of the aforementioned derivatives has been described in earlier works of the research team [27,28,34].

### 2.1. 0.02. Ac-H and 0.1Ac-H

Acetylated starch samples with degrees of substitution (DS) of 0.02 and 0.1 were subjected to hydrolysis utilizing BAN 480L as the hydrolytic enzyme preparation. This hydrolysis was conducted in a continuous recycle membrane reactor (CRMR), featuring a membrane with a molecular weight cut-off of 50 kDa. The hydrolysis utilized 5% (*w/w*) starch solutions that were prepared through gelatinization and subsequent sterilization. The enzyme was administered at a concentration of 0.1 mL per 7 liters of starch solution. The hydrolysis process was executed over 100 min, during which ultrafiltration (UF) separation was performed at a transmembrane pressure of 0.1 MPa. A comprehensive account of the hydrolysis parameters and selected physicochemical properties of the resultant preparation is provided in reference [34].

### 2.2. Ac-FD-H

A commercial food-grade double-modified starch, specifically acetylated distarch phosphate, was subjected to hydrolysis. The pre-hydrolysis phase occurred concurrently with the gelatinization of a 20% starch suspension, which contained 0.25 mL of Termamyl Supra (Novozymes, Frederiksberg, Denmark) per kilogram of starch. This gelatinization process was conducted for 60 min at a temperature of 85 °C, accompanied by continuous stirring. Subsequently, the starch paste was sterilized at 121 °C for 20 min.

The principal hydrolysis was executed in a continuous recycle membrane reactor (CRMR), characterized by a molecular weight cut-off of 50 kDa. The reactor was charged with the product obtained from the pre-hydrolysis phase, and hydrolysis was performed utilizing an ultrafiltration (UF) membrane separation process over 120 min at a temperature of 60 °C. This was conducted under a transmembrane pressure of 0.15 MPa, with an enzyme concentration of 0.3 mL of the preparation BAN 480L (Novozymes, Frederiksberg, Denmark) per kilogram of starch. The reaction mixture’s volume was meticulously regulated by the automated systems of the BioFlo (New Brunswick Scientific Co., Inc., Enfield, CT, USA) reactor. A comprehensive overview of the hydrolysis process parameters and selected physicochemical properties of the resulting preparation is detailed in reference [27].

### 2.3. Ac-Ox-H

A commercial food-grade oxidized starch, characterized by a carboxyl group content of 0.04%, underwent further acetylation to yield a doubly modified starch with a degree of acetylation of 0.1. The hydrolytic enzyme used in this process was BAN 480L, applied at a concentration of 0.3 mL per kilogram of solid substrate. The primary hydrolysis was conducted in a continuous recycle membrane reactor (CRMR). The membrane separation, concurrent with the hydrolysis, was executed in one of two ultrafiltration (UF) units equipped with ceramic membranes having molecular weight cut-offs of 15 kDa or 8 kDa. This membrane filtration process was maintained at a transmembrane pressure of 0.5 MPa and continued for 2 h. A detailed account of the hydrolysis parameters and selected physicochemical properties of the resulting preparation is provided in reference [28].

In addition, the following reagents were used in the research: CTAB—hexadecyltrimethylammonium bromide, SDS—sodium dodecyl sulfate, Triton™ X-100—polyethylene glycol tert-octylphenyl ether and toluene, produced by Merck (Burlington, MA, USA); Akofine P™—fully saturated, deodorized vegetable oil produced by AAK (Karlshamn, Sweden); and MAG—1-(7Z-tetradecenoyl)-rac-glycerol, produced by Avanti Research (Alabaster, AL, USA).

The equilibrium surface and interfacial tension measurements were conducted using the Du Noüy ring method. The investigations employed a K12 tensiometer from Krüss (Hamburg, Germany), equipped with a Du Noüy ring. The dynamic surface tension was measured by the drop shape analysis technique, employing a tensiometer TRACKER™ (Teclis Scientific, Civrieux d’Azergues, France). All measurements were conducted at a temperature of 21 °C ± 0.1 °C. The standard deviation of the surface/interfacial tension measurements was approximately 0.05 mN/m.

The experimentally determined surface tension isotherms (γ = f(c)) were approximated by the Szyszkowski isotherm equation, where γ_0_ is the surface/interfacial tension for concentration *c* = 0; and *A_Sz_* and *B_Sz_* are the Szyszkowski coefficients [34]:

(1) $$\gamma_{i}=\gamma_{0}\left[1-B_{Sz}\ln(\frac{c}{A_{Sz}}+1)\right]$$

Differentiating the above equation against the concentration and introducing the expression *dγ/dc* into the Gibbs isotherm (in which *R*—gas constant, *T*—temperature):

(2) $$\Gamma =-\frac{c}{RT}\frac{d\gamma }{dc}$$

The dependence of the surface excess as a function of concentration was obtained:

(3) $$\Gamma^{Sz}=\frac{\gamma_{0}B_{Sz}}{RT}\frac{c_{i}}{\left(c_{i}+A_{Sz}\right)}$$

Based on the coefficients of the Szyszkowski equation, the free energy of adsorption (ΔG_ads_) and the value of the surface concentration at the saturated interface (Γ_∞_) were estimated:

(4) $$\Delta G_{\mathrm{ads}}=-RTlnA_{Sz}$$

(5) $$\Gamma_{\infty }=\frac{\gamma_{0}B_{Sz}}{RT}$$

Based on the surface concentration value Γ_∞_, A_min_ was estimated, i.e., the amount of surface area occupied by a single statistical molecule in the monomolecular layer at the saturated interface, where *N_A_* is the Avogadro’s number (*N_A_*= 6.023 × 10^23^):

(6) $$A_{\min}=\frac{1}{\Gamma_{\infty }N_{A}}$$

The molar surface fractions in the mixed adsorption layer, *X_i_*, were calculated using the following equation:

(7) $$\frac{{X_{1}}^{2}\ln(\frac{C_{1,t}}{{C_{1,t}}^{0}X_{1}})}{{\left(1-X_{1}\right)}^{2}\ln[\frac{C_{2,t}}{{C_{2,t}}^{0}(1-X_{1})}]}=1$$

In the equation, *C_1,t_ (=α·C_t_)* and *C_2,t_ (=(1 − α)C_t_)* represent the total molar concentrations of individual surfactant components 1 and 2 in the volume phase containing the binary mixture, which causes the reduction in surface tension in the examined system to a specific value γ_i_. Meanwhile, *C^0^_1,t_* and *C^0^_2,t_* denote the total molar concentrations of individual surfactant substances 1 and 2 in the volume phase that produce the same surface tension value γ_i_ as in the mixed system.

Following the established methodology, emulsions were prepared by weighing the necessary proportions of the water and oil phases in a mass ratio of 1:4. Each phase was heated separately in a water bath to 70 °C while being continuously mixed with a mechanical stirrer (Heidolph, Schwabach, Germany, model number: RZR 2021). Once heated, the stirrer speed was increased to a constant 500 rpm. The water phase mixture was then added to the oil phase mixture slowly and continuously to create a water-in-oil (W/O) emulsion. Heating was then stopped, allowing the system to cool naturally. Emulsification continued until the system reached room temperature, approximately 25 °C, while maintaining the same stirring speed. The resulting emulsions were stored at room temperature for further stability studies.

The stability of the emulsion systems was assessed using a Turbiscan LAB Expert (Formulaction, Toulouse, France) device, which operates based on the measurement of light intensity that is both backscattered and transmitted through the sample.

A list of all abbreviations used can be found before the References section.

## 3. Results and Discussion

### 3.1. Dynamic Surface Tension

**Figure 1:**
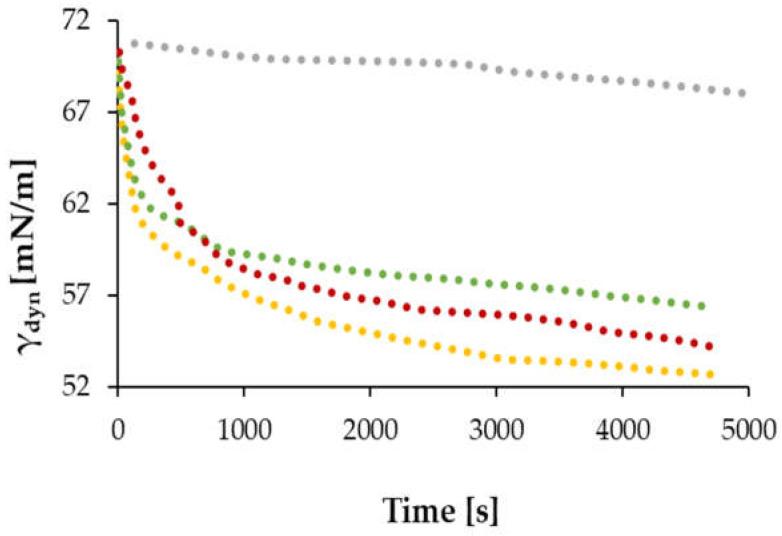
Dynamic surface tension in the air/water system over time for (●) 0.1Ac-H, c = 0.01%; (●) 0.02Ac-H, c = 0.01%; (●) 0.02Ac-H, c = 0.1%; and (●) Ac-FD-H, c = 0.1%.

Figure 1 compares the dynamic surface tension isotherms in an air/water system for cross-linked acetylated starch phosphate (Ac-FD-H) hydrolysate and acetylated starch hydrolysates with different degrees of substitution, which were described in our previous paper [34]. As can be easily seen, an additional cross-linking of the acetylated derivative significantly changes the surface activity of the starch preparation. The observed reduction in the ability to lower surface tension is most likely affected by the fact that the cross-linking process leads to the formation of bridges between macromolecules, which changes their tendency to adsorb at the interface.

**Figure 2:**
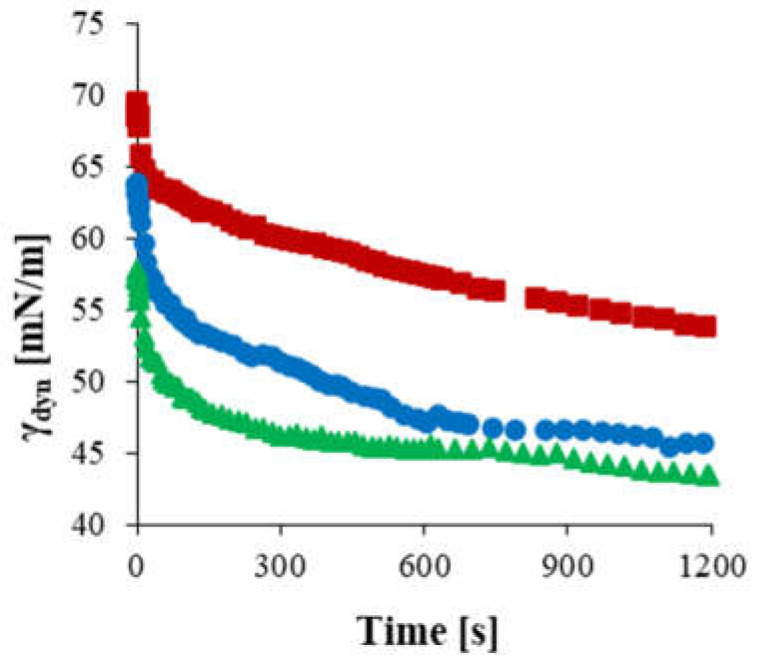
Dynamic surface tension in the air/water system over time for hydrolysates obtained at (■) 90, (●) 120, and (▲) 180 min of the oxidized acetylated starch hydrolysis process (Ac-Ox-H) conducted in a CRMR with membranes having cut-offs of 15 and 8 kDa.

As stated, the conditions of the hydrolysis process also influence the surface activity of the obtained starch products. Figure 2 illustrates the changes in dynamic surface tension measured for the components of the filtrate fractions obtained during the hydrolysis of double-modified starch preparations, involving oxidation and acetylation in a CRMR.

During the hydrolysis of acetylated oxidized starch, the surface activity of the components increased with the progression of the process. The fractions collected at the final stage of the hydrolysis process, i.e., at 180 min, exhibited the highest surface tension reduction capacity. An analysis of the dynamic surface tension curves indicated that the use of a membrane with a low separation capability, 8 kDa, resulted in the separation of maltodextrins with relatively high DE values, which were characterized by high surface activity.

### 3.2. Surface Activity in Model Binary Systems: Modified Starch Hydrolysate and Surfactant

Starch exhibits the ability to form complexes with amphiphilic substances. These complexes influence the starch retrogradation process, which has significant practical implications.

**Figure 3:**
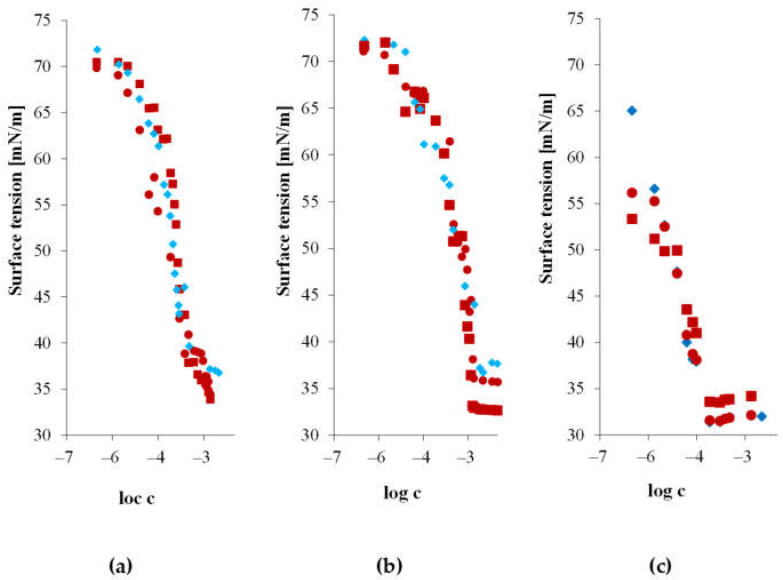
Surface tension as a function of the logarithm of molar concentration for aqueous solutions: (♦) surfactant, (■) mixture of surfactant and 0.02Ac-H hydrolysate, and (●) mixture of surfactant and 0.1Ac-H hydrolysate with a molar ratio of 1:6 for (**a**) CTAB, (**b**) SDS, and (**c**) Triton X-100.

In the subsequent stage of research conducted as part of this study, the behavior of the obtained modified starch hydrolysates in the presence of surfactants was analyzed. To determine the effect of the type of added surfactant on the magnitude and nature of interactions with the starch preparation, surface activity measurements were carried out for a series of binary systems, including starch hydrolysate and a model surfactant (Figure 3).

Adding 1% of either 0.02Ac-H or 0.1Ac-H acetylated starch hydrolysate to aqueous surfactant solutions resulted in only a slight change in the surface activity of the mixed systems. In all cases, the mixtures showed synergistic effects in reducing surface tension in the air/water system. The changes in surface tension of the mixed systems were influenced by the type of surfactant used and the degree of esterification of the starch hydrolysate. However, regardless of the type of surfactant present, the degree of esterification of the starch hydrolysate consistently affected the level of synergy in surface tension reduction. Mixtures containing the 0.02Ac-H acetylated starch derivative demonstrated greater surface activity compared to the binary systems with the 0.1Ac-H acetylated starch hydrolysate, which has a higher degree of acetylation.

The type of surfactant also played a significant role. Depending on the nature of the surfactant, different shifts in the CMC point for the mixed system were observed. Comparing the estimated CMC values obtained from the graphs for single-component surfactant systems and mixed systems allowed for the assessment of the impact of adding the hydrolysate on the shift in the micellization region in the starch system.

For the system with the cationic surfactant CTAB, a shift in the critical micelle concentration (CMC) of the mixture towards higher concentrations was observed. For the single-component system, i.e., the CTAB solution, the CMC was 0.61 mmol/dm^3^. After the addition of the starch hydrolysate with a degree of substitution (DS) of 0.02, the CMC increased to 0.96 mmol/dm^3^. The addition of the 0.1Ac-H hydrolysate caused a shift in the CMC towards higher concentrations to a value of 0.89 mmol/dm^3^.

The addition of starch hydrolysate to an aqueous solution of anionic surfactant caused a shift in the CMC towards lower concentrations. The CMC for the SDS solution was 7.08 mmol/dm^3^. The addition of the 0.02Ac-H hydrolysate reduced the micellization concentration to 5.37 mmol/dm^3^. Similarly, the system with the 0.1Ac-H hydrolysate decreased the critical micelle concentration to 5.01 mmol/dm^3^.

In contrast to the mixtures with cationic and anionic surfactants, the mixed system with the nonionic surfactant showed a different behavior. The addition of starch hydrolysate to the Triton X-100 solution caused only a slight change in the CMC towards lower concentrations, regardless of the degree of substitution of the hydrolysate.

The CMC values for aqueous surfactant solutions and mixed systems are summarized in Table 1.

In the case of mixtures with ionic surfactants, the shift in CMC is accompanied by an increase in surface activity within the micellization region.

#### 3.2.1. Adsorption Parameters for Binary Systems: Acetylated Starch Hydrolysate and Surfactant

For the study of surface activity in binary systems, acetylated starch hydrolysates with degrees of acetylation of 0.02 and 0.1 were selected. The method for obtaining these derivatives is detailed in [34]. Three types of surfactants were analyzed: the cationic hexadecyltrimethylammonium bromide (CTAB), the anionic sodium dodecyl sulfate (SDS), and the nonionic Triton^®^ X-100. Mixtures with a molar ratio of starch to surfactant of 6:1 were investigated.

The resulting surface tension isotherms as a function of the logarithm of molar concentration for the studied mixed systems are shown in Figure 3.

The experimentally determined surface tension isotherms for surfactants and binary mixtures of starch hydrolysate and surfactant were approximated using the Szyszkowski Equation (1) [34]. Based on the Szyszkowski coefficients A_Sz_ and B_Sz_, the adsorption parameters for the studied systems were estimated, including the free energy of adsorption ΔG_ads_, the surface excess Γ_∞,_ and the area occupied by a single statistical molecule in the saturated adsorption layer A_min_.

Based on the regular solution theory and using the Rossen Equation (7), the surface molar fractions X_i_ at the aqueous/air phase boundary were estimated from the isotherms of the mixed systems (Table 1).

In all the studied binary systems, the proportion of acetylated starch hydrolysate at the saturated aqueous/air phase boundary was estimated to be between 0.31 and 0.42. Thus, the composition of the bulk phase was significantly different from that of the adsorption layer. The excess of acetylated starch hydrolysate in the mixed solutions was 6 mol/mol, whereas at the air/water interface, the hydrolysate molecules occupied about 40% of the interfacial area.

The adsorption capacity in the binary systems of starch hydrolysate and surfactant was greater than the surfactants alone. However, the effectiveness of adsorption, expressed as the surface excess at the saturated interphase (Γ_∞_), was higher for all three surfactants compared to the binary systems of hydrolysate and surfactant (Table 1). Analyzing the values of the free energy of adsorption (ΔG_ads_), it can be noted that for all considered mixed systems, the tendency for adsorption at the air/water interface was greater than for the studied surfactants.

Hossail and Mondal [35] who studied solutions of starch derivatives (potato starch acetate/octenylsuccinate and acetate/dodecenylsuccinate) in the presence of ionic (SDS, CTAB) and nonionic surfactants (TWEEN 20) showed that surface and interfacial tensions for starch–surfactants mixtures were lower than those for only starch, particularly at lower concentrations. In the opinion of the cited authors, starches are surface-inactive. However, due to H-bonding with ionic surfactants, they became more surface-active, which lowers the value of surface tension. Furthermore, the cited publication, similar to our results, indicates a minimal dependence of the measured surface or interfacial tensions on both the degree of substitution of modified starch and the amylose content of starch.

Radhika and Moorthy studied the interactions in solutions containing starch and added surfactants [36]. The authors proved the complexation of cassava starch with different surfactants, and furthermore, it was found that the production of starch–surfactant complex is easier compared to cross-linking by chemical reactions.

Studies on mixtures of modified starches with surfactants were also conducted by Zang et al. [37], who found that the formation of inclusion complexes with linear surfactants like SDS might provide a new means of stabilizing hydrophobically modified starch nanoparticles in water.

#### 3.2.2. Surface Activity of Binary Systems Consisting of Cross-Linked Starch Hydrolysate–Surfactant

**Figure 4:**
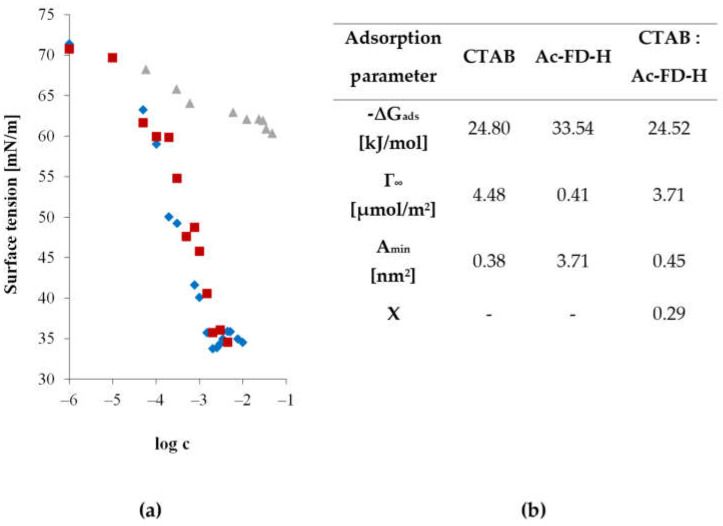
Surface activity: (**a**) Surface tension as a function of the logarithm of molar concentration for aqueous solutions: (♦) surfactant, (▲) Ac-FD-H hydrolysate, and (■) mixture of surfactant and Ac-FD-H hydrolysate with a molar ratio of 1:6; (**b**) adsorption parameters.

The surface activity of a mixture containing starch hydrolysate with a low degree of cross-linking, specifically acetylated distarch phosphate (Ac-FD-H), and CTAB, a model cationic surfactant, at a molar ratio of starch to surfactant of 6:1, was studied. The surface tension isotherms as a function of the logarithm of the molar concentration for the examined mixed systems are presented in Figure 4a, while the adsorption parameters and surface mole fractions are summarized in Figure 4b.

In binary systems with a cationic surfactant, no synergism was observed in reducing the surface tension. The addition of a cross-linked starch hydrolysate to a CTAB solution resulted in the deterioration of the adsorption properties of the mixture. Consequently, a slight increase in the surface tension of the system was observed. It should also be noted that the adsorption efficiency of the binary Ac-FD-H and CTAB system was lower than that of the pure CTAB solution. Furthermore, no shift in the critical micelle concentration (CMC) was observed for the CTAB and Ac-FD-H hydrolysate mixture.

An analysis of the composition of the mixed adsorbed layers shows that in the case of mixtures, surfactant molecules dominate in the saturated surface layer. The contribution of acetylated cross-linked starch hydrolysate molecules in the adsorbed monolayer was minimal, at only 20%.

#### 3.2.3. Surface Activity of Binary Systems Consisting of Acetylated and Oxidized Starch Hydrolysate and Surfactant

The effect of adding the hydrolysate of potato starch modified through both oxidation and acetylation (Ac-Ox-H hydrolysate with a low content of carboxyl (0.04) and acetyl groups (0.02), obtained through enzymatic hydrolysis using CRMR and two-stage ultrafiltration on membranes with cut-offs of 50 and 15 kDa) on the adsorption properties of a binary system of starch hydrolysate and cationic surfactant was investigated. Mixtures with a molar ratio of hydrolysate to surfactant of 6:1 were studied.

**Figure 5:**
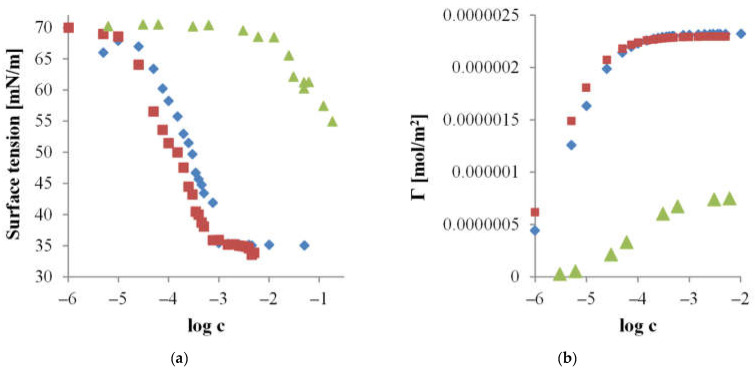
Surface tension as a function of the logarithm of molar concentration (**a**), and surface excess as a function of the logarithm of molar concentration (**b**) for aqueous solutions of (♦) CTAB, (▲) Ac-Ox-H hydrolysate, and (■) a mixture of CTAB and Ac-Ox-H hydrolysate at a molar ratio of 1:6.

The experimentally obtained surface tension isotherms as a function of the logarithm of molar concentration for the examined mixed system are presented in Figure 5a. The surface excess values, estimated for the investigated composition and individual components, are illustrated in Figure 5b.

The addition of Ac-Ox-H hydrolysate to aqueous solutions of the cationic surfactant CTAB causes a slight increase in the surface activity of the mixed system. The binary mixture thus exhibits synergism in reducing surface tension at the air/water interface, as well as synergism in micelle formation. In the binary system, a shift in the critical micelle concentration (CMC) towards lower concentrations was observed compared to the single-component system with surfactant alone.

The free energy of adsorption at the air/water interface (Table 2) estimated for the binary mixture was similar to the ΔG_ads_ for the surfactant present in the mixture. Similarly, the adsorption efficiency for the hydrolysate mixture, expressed as the surface excess at the saturated phase boundary, was comparable to the adsorption efficiency of CTAB. In both cases, the value of Γ^∞^ was 2.3 μmol/m^2^.

Additionally, as shown in Figure 4b, for the binary system, a saturation of the adsorbed layer occurred at a lower bulk concentration than for the hydrolysate solution. This indicates that the addition of a small amount of cationic surfactant to the acetylated and oxidized starch hydrolysate solution allows for a significantly more efficient saturation of the air/water surface layer.

The characteristics of the obtained surface tension isotherms indicate that the studied starch preparations were multi-component substances. The addition of a solution of modified starch hydrolysate to aqueous solutions of the surfactants generally led to an increase in the surface activity of the mixed system. Therefore, the mixtures exhibited synergism in reducing surface tension at the air/water interface. The magnitude of the change in surface tension depended both on the type of surfactant used and on the type of starch hydrolysate.

The investigated mixtures also showed synergism in micelle formation. In most cases, a shift in the critical micelle concentration (CMC) of the binary systems (hydrolysate) towards lower concentrations was observed, which is of particular applicative significance. The addition of a small amount of surfactant to a given starch hydrolysate (at a molar ratio of 1:6) can contribute to a reduction in the amount of starch hydrolysate used, for example, in the formulation of dispersions.

#### 3.2.4. Surface Activity of Binary Systems Consisting of Modified Starch Hydrolysate–Glucopone

The food industry offers a wide range of high-quality, biodegradable products free of pathogens and toxins. Alkyl polyglucosides belong to this group of compounds. These are nonionic surfactants known commercially as Glucopone. Alkyl polyglucosides interact with starch to form complexes [38]. According to reports from the literature, in the presence of starch, an increase in surface tension occurs compared to the solution of the surfactant alone. Thus, starch, which generally does not exhibit surface activity, can influence the surface tension of a two-component mixture when forming a complex with a nonionic compound.

For the study of surface activity in binary systems of the type hydrolysate–Glucopone, two hydrolysates of acetylated starch with different degrees of substitution, namely 0.02Ac-H and 0.1Ac-H, and a hydrolysate of cross-linked starch, i.e., acetylated distarch phosphate (Ac-FD-H), were selected. Two types of nonionic surfactants were analyzed: Glucopone^®^215 and Glucopone^®^600. Mixtures with a molar ratio of starch to surfactant of 6:1 were examined.

**Figure 6:**
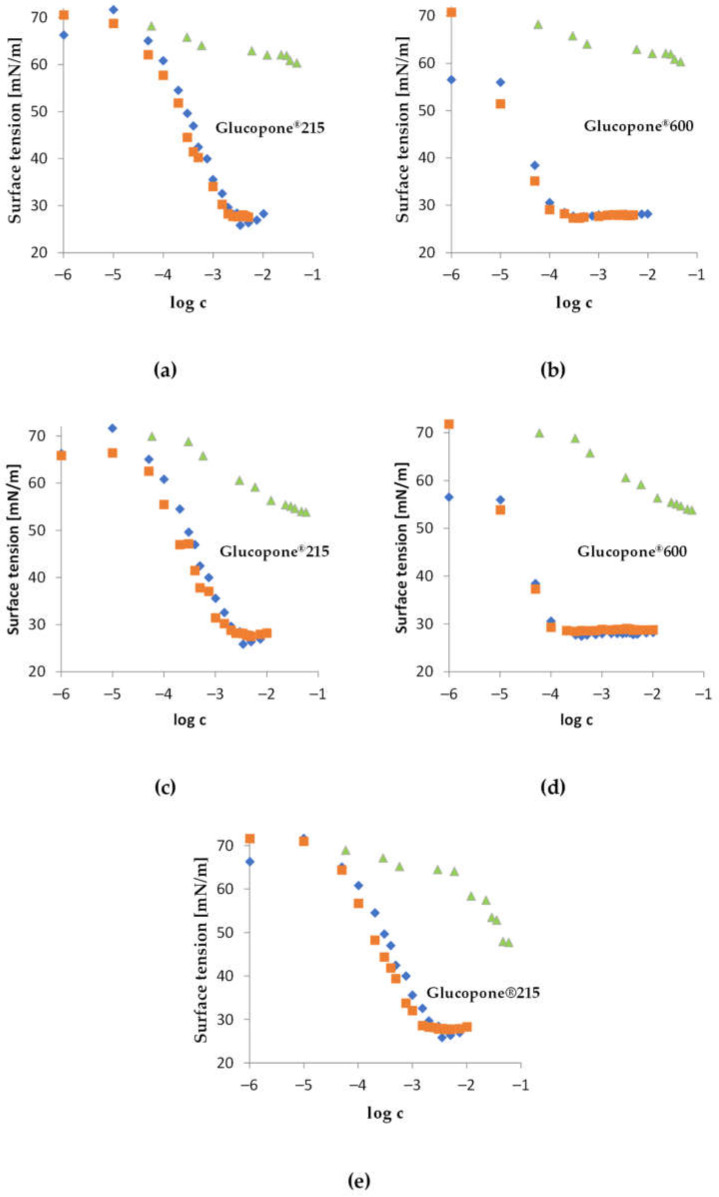
Surface tension as a function of the logarithm of molar concentration for aqueous solutions of (♦) Glu, (▲) hydrolysate, and (■) a mixture of surfactant and hydrolysate, at a molar ratio of 1:6 for (**a**) and (**b**) Ac-FD-H; (**c**) and (**d**) 0.02Ac-H; and (**e**) 0.1Ac-H.

The surface tension isotherms as a function of the logarithm of the molar concentration obtained for the studied mixed systems are presented in Figure 6.

The analysis of the surface tension isotherms presented in Figure 6 indicates the formation of complexes between the surfactant and the hydrolysate; however, it does not suggest the occurrence of synergism in reducing the minimum surface tension. The γ_CMC_ values for the mixed systems under consideration, as well as for solutions of Glukopon^®^215 and Glucopone^®^600, were identical. Furthermore, the addition of cross-linked starch hydrolysate Ac-FD-H to the Glucopone^®^600 solution resulted in a slight decrease in the surface activity of the two-component system.

The commercial products Glucopone^®^215 and Glukopone^®^600 exhibited a similar ability to reduce surface tension, with γ_min_ in both cases reaching approximately 28 mN/m. However, the critical micelle concentration (CMC) for Glucopone^®^215 was around 2.5 mmol/dm^3^, while for the second surfactant, Glucopone^®^600, the CMC was almost ten times lower, at approximately 0.2 mmol/dm^3^. The surfactants studied also showed significantly different tendencies toward adsorption at the air/water interface. The free energy of adsorption for Glucopone^®^215 and Glucopone^®^600 was −26.6 kJ/mol and −48.9 kJ/mol, respectively.

In most of the binary systems studied, a shift in the CMC of the mixtures toward lower concentrations was observed compared to the solutions of the individual surfactants. However, the extent of the CMC shift in the mixed systems depended on the type of surfactant used. Mixtures containing Glucopone^®^215 caused a significantly larger CMC shift than mixtures containing Glucopone^®^600. The exception was the Ac-FD-H–Glucopone^®^600 system, in which a slight shift in the CMC toward higher concentrations was noted.

Table 3 summarizes the adsorption parameters estimated for the individual compounds and their binary mixtures.

The addition of modified starch hydrolysate reduced the adsorption tendency of binary systems with Glucopone^®^600. An opposite adsorption tendency at the air/water interface was observed for mixtures of hydrolysates with Glucopone^®^215. The inclusion of modified starch hydrolysate increased the adsorption tendency of the binary system compared to the single-component surfactant system. The lowest values of the free energy of adsorption were estimated for binary systems containing acetylated starch hydrolysates, while the adsorption tendency involving cross-linked starch hydrolysate was slightly lower.

The mole fraction of the starch hydrolysate in the saturated adsorption monolayer differed from that in the bulk phase. The surface mole fraction of the hydrolysate ranged from 0.40 to 0.47. It is also important to note that the surface mole fraction of starch hydrolysates containing acetyl groups was higher in the case of cross-linked starch.

The determined values of the intermolecular interaction parameter (β) in the mixed systems considered were negative, indicating an attractive nature of interactions between the adsorbed molecules. It should also be noted that the intermolecular interactions between Glucopone^®^600 molecules and modified starch hydrolysate molecules were almost three times stronger than the attractive interactions between Glucopone^®^215 molecules and hydrolysate molecules.

The interactions between modified starch hydrolysate molecules and alkyl polyglucoside molecules depend on both the type of surfactant and the type of starch hydrolysate. It was found that the addition of starch hydrolysate to the surfactant solution caused a minimal increase in surface tension, which is consistent with the observations reported by V. Bravo Rodriguez et al. [39]. However, the CMC shift for mixtures containing Glucopone and soluble starch, as described in the cited work, occurred toward higher concentrations compared to the systems studied in this work with modified starch hydrolysate and polyglucoside. Therefore, the interactions between the components of the mixture depend on the type of starch modification.

### 3.3. Interfacial Activity in Model Oil/Water System

**Figure 7:**
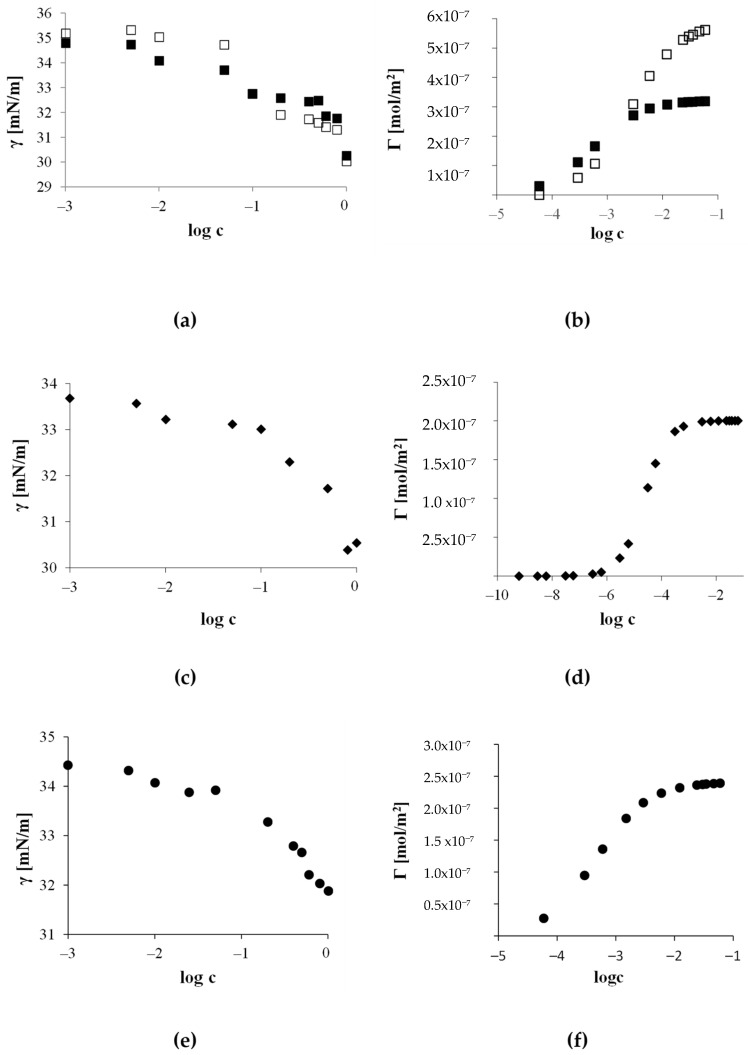
Interfacial tension as a function of the logarithm of concentration (**a**,**c**,**e**) and surface excess as a function of the logarithm of concentration (**b**,**d**,**f**) for aqueous solutions of modified starch hydrolysates in the toluene–water system for (■) 0.02Ac-H, (□) 0.1Ac-H, (♦) Ac-FD-H, and (●) Ac-Ox-H.

Interfacial tension measurements were conducted at the toluene–water interface for potato starch derivatives (Figure 7). Surface activity at the oil/water interface plays a crucial role in the stability and characteristics of emulsion systems. In such systems, immiscible oil and water phases can be stabilized by the presence of surfactants, which reduce the interfacial tension, facilitating the dispersion of one phase into the other and thereby stabilizing the emulsion.

An analysis of the curves presented in Figure 7a,b indicates that the ability to reduce interfacial tension in the toluene–water system for derivatives 0.02Ac-H and 0.1Ac-H is very similar. However, there is a difference in adsorption efficiency, expressed as the surface excess at the saturated interface. The derivative with a higher degree of substitution by acetyl groups, namely 0.1Ac-H, forms a more densely packed layer at the interface compared to the lower substituted derivative. The surface excess concentration for the 0.1Ac-H hydrolysate, equal to 0.59 μmol/m^2^, is twice as high compared to the Γ_∞_ value estimated for the 0.02Ac-H derivative. However, the concentration at which phase saturation occurred is about an order of magnitude higher for the derivative with the higher degree of substitution (DS). This implies that the 0.1Ac-H hydrolysates form a relatively densely packed adsorption layer, but only if the solution concentration is not less than approximately 10^−2^ mol/dm^3^.

The 0.02Ac-H hydrolysate shows a greater tendency for adsorption in the toluene–water system (Table 4). Simultaneously, the surface area occupied by its statistical molecule at the saturated interface is almost twice as large as the Γ_∞_ for the 0.1Ac-H derivative.

The experimentally determined interfacial tension isotherm as a function of the logarithm of molar concentration and the calculated surface excess values as a function of the logarithm of molar concentration for the cross-linked hydrolysate Ac-FD-H are compiled in Figure 6c,d. The quantitative characterization of the adsorption process for acetylated cross-linked starches is presented in Table 4.

The starch hydrolysate Ac-FD-H exhibits the highest tendency for adsorption in the toluene–water system (−∆G_ads_ = 26.11 kJ/mol) among all analyzed derivatives, simultaneously reducing the interfacial tension to a value of 30.5 mN/m at a 1% concentration. Additionally, as shown in Figure 6d, in this system, the saturation of the interface is achieved at the lowest concentrations, namely 0.6 μmol/dm^3^. The packing density of the adsorption layer with Ac-FD-H molecules is 2 × 10^−7^ mol/m^2^. Saturation of the adsorption layer with cross-linked hydrolysate molecules occurs at the lowest molar concentrations. Moreover, the surface area occupied by the average adsorbed molecule at the toluene–water interface (A_min_) is the largest for the cross-linked derivative, measuring 8.14 nm^2^. This indicates a distinct orientation of the adsorbed molecules at the interface for the analyzed derivatives.

A 1% aqueous solution of the hydrolysate Ac-Ox-H (Figure 7e,f) reduces the interfacial tension to 31.8 mN/m and exhibits a similar tendency for adsorption as the acetylated derivative with a low degree of functional group substitution (Table 4).

### 3.4. Model Emulsion

As demonstrated in the previous subsections, starch hydrolysates obtained from enzymatic hydrolysis of chemically modified derivatives exhibit surface activity in both air/water and toluene–water systems. The effectiveness in reducing surface and interfacial tension varies depending on the type of modification and the degree of substitution with specific functional groups. The process of dual modification, namely oxidation and acetylation, leads to an increase in surface activity, thus double-modified derivatives show a greater surface activity compared to native starch. Additionally, enzymatic hydrolysis further enhances the ability of the preparations to reduce surface and interfacial tension. Differences in the surface activity of the studied biopolymers are indicated by adsorption parameters, which allow for a quantitative assessment of surface activity.

As part of preliminary application studies for the obtained hydrolysates, tests were conducted to assess their suitability for stabilizing model emulsions.

Reports from the literature indicate that acylglycerols, particularly monoacylglycerols, are effective emulsifiers [38,40,41,42]. Therefore, emulsions of the W/O type were created with the addition of monoacylglycerol as the emulsifier. The use of monoacylglycerol (MAG) in the formulation of the emulsions resulted in dispersions with stability exceeding 50%.

The stability of the resulting emulsion systems was evaluated based on the results of measurements of the intensity of the light backscattered by the test sample. The light source was an electroluminescent diode emitting near-infrared light at 880 nm.

The curves obtained show the intensity of the backscattered light (%) as a function of the height of the measuring cell (mm). These profiles provide a macroscopic image of the sample at a given time. This measurement allows, among other things, for the detection of creaming and sedimentation phenomena over time, which can be used to analyze the kinetics of emulsion destabilization phenomena.

**Figure 8:**
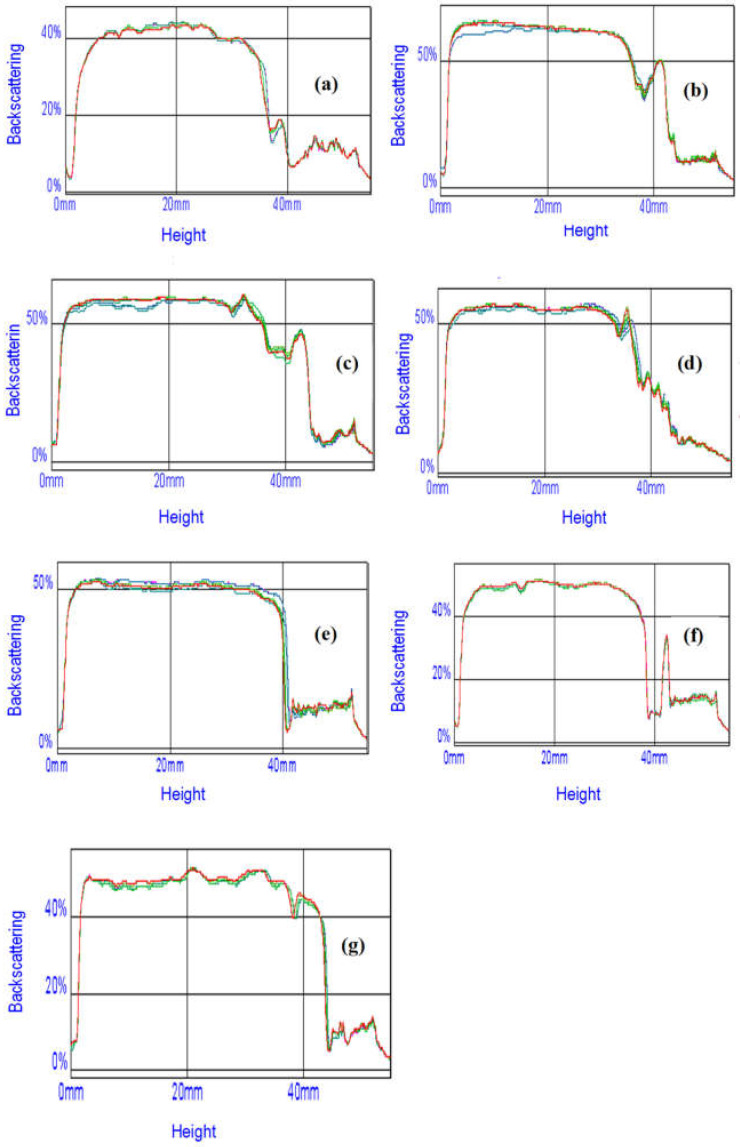
Stability of W/O emulsions composed of soybean oil, water, hydrogenated oil Akofine, starch hydrolysate, and MAG, in mass ratios of MAG to starch hydrolysate as follows: (**a**) 0:10, (**b**) 7:3, (**c**) 5:5, (**d**) 4:6, (**e**) 3:7, (**f**) 2:8, and (**g**) 1:9, after: (**―**) 0, (**―**) 3, (**―**) 6, (**―**) 9, and (**―**) 12 days.

A series of emulsions were produced consisting of soybean oil, water, and double-modified starch hydrolysate, by oxidation and acetylation (Ac-Ox-H); a modifier of the oil phase, namely hydrogenated oil Akofine; and monoacylglycerol (MAG). To determine the effect of the starch preparation on emulsion stability, several dispersions with varying amounts of hydrolysate and monoacylglycerol were prepared. Emulsion stability readings were taken daily over a period of 12 days. The data obtained from these measurements are presented in Figure 8.

An analysis of the curves presented in Figure 8 reveals a decrease in the stability of the emulsion system with an increase in the proportion of starch hydrolysate as the emulsifier, or alternatively, a decrease in the proportion of MAG in the formulated system. The emulsion system with MAG completely replaced by the starch preparation (Figure 8a) exhibited the lowest stability. However, the addition of modified starch hydrolysate up to approximately 50–60% did not cause a significant deterioration in emulsion stability. It should also be noted that all the created dispersions remained stable over time. Preliminary studies on the application potential of modified starch hydrolysates demonstrated that they could serve as components of emulsifiers/stabilizers, contributing, for instance, to the production of completely biodegradable emulsion systems.

## 4. Conclusions

The dynamic surface tension measurements on acetylated starch hydrolysates in an air/water system allow us to analyze how the concentration and degree of substitution with acetyl groups affect the adsorption dynamics of the starch derivatives. We stated that the diffusion coefficients, calculated from the experimentally determined dynamic surface tension isotherms, are influenced by the degree of acetylation of the hydrolysate and increase with the concentration of the solution.

In all the systems we studied containing acetylated starch hydrolysate and a model surfactant, we observed synergistic effects in reducing surface tension at the air/water interface. The changes in surface tension of the mixed systems were affected by the type of surfactant used and the degree of esterification of the starch hydrolysate. We consistently observed that the degree of esterification of the starch hydrolysate affected the level of synergy in surface tension reduction, regardless of the type of surfactant used. However, different shifts in the CMC point for the mixed systems were observed depending on the nature of the surfactant.

Our studies showed that the composition of the bulk phase was significantly different from that of the adsorption layer in the binary systems we studied. The adsorption capacity in the mixed starch hydrolysate and surfactant systems was greater than in the systems with surfactants alone. However, the effectiveness of adsorption, expressed as the surface excess at the saturated interphase (Γ_∞_), was higher for all three surfactants compared to the binary systems. Additionally, the tendency for adsorption at the air/water interface was greater for the studied surfactants.

When we examined binary systems containing CTAB as a model cationic surfactant and cross-linked starch hydrolysate, we observed no synergism in reducing surface tension and CMC, and found that the surfactant molecules dominated at the saturated surface layer.

We also found that adding a hydrolysate of potato starch doubly modified through both oxidation and acetylation to aqueous solutions of the cationic surfactant CTAB creates a mixture that demonstrates synergy in reducing surface tension at the air/water interface and in micelle formation. Even a small amount of cationic surfactant added to the acetylated and oxidized starch hydrolysate solution significantly increased the efficiency of saturation of the air/water surface layer.

The interaction of acetylated starch with different degrees of substitution and cross-linked starch (acetylated distarch phosphate) with alkyl polyglucoside molecules depends on the type of polyglucoside used and the type of starch modification. We observed that adding modified starch hydrolysate reduced the tendency for adsorption in mixtures with Glucopone^®^600. However, we observed the opposite adsorption tendency at the air/water interface for mixtures of the considered hydrolysates with Glucopone^®^215.

Our study of the impact of starch preparation on emulsion stability with different proportions of hydrolysate and monoacylglycerol indicated that adding modified starch hydrolysate up to about 50–60% did not significantly reduce emulsion stability. Importantly, all the dispersions we created remained stable over time. Our initial studies on the potential application of modified starch hydrolysates indicated that they could be used as components of emulsifiers and stabilizers, contributing to the production of completely biodegradable emulsion systems.

## Figures and Tables

**Table 1:** Adsorption parameters of mixed systems consisting of acetylated starch hydrolysate and surfactant at a molar ratio of 6:1.

	Adsorption Parameters	−∆G_ads_ [kJ/mol]	Γ_∞_ [mol/m^2^]	A_min_ [nm^2^]	X (γ = 60mN/m)	CMC mmol/L	γ_CMC_ mN/m
Solution							
Single-component system	SDS	19.63	5.50	0.40	-	7.08	37.77
	CTAB	24.80	4.48	0.38	-	0.61	36.78
	TRITON	42.03	1.56	1.07	-	0.25	31.99
	0.02Ac-H	22.76	1.17	1.42	-		
	0.1Ac-H	15.63	2.58	0.65	-		
Binary system	SDS + 0.02Ac-H	23.55	3.06	0.54	0.42	5.37	33.10
	SDS + 0.1Ac-H	23.33	2.79	0.60	0.42	5.01	35.85
	CTAB + 0.02Ac-H	24.29	3.62	0.46	0.35	0.96	35.81
	CTAB + 0.1Ac-H	30.44	2.18	0.76	0.41	0.89	34.44
	TRITON + 0.02Ac-H	47.50	1.23	1.35	0.33	0.25	31.99
	TRITON + 0.1Ac-H	43.29	1.53	1.08	0.31	0.25	32.08

**Table 2:** Adsorption parameters of mixed systems consisting of acetylated starch hydrolysate–surfactant at a molar ratio of 6:1.

Parameter	Unit	CTAB	Ac-Ox-H	CTAB: Ac-Ox-H
Γ_∞_	μmol/m^2^	2.32	0.76	2.30
A_min_	nm^2^	0.72	2.19	0.72
−∆G_ads_	kJ/mol	30.32	23.16	31.40

**Table 3:** Adsorption parameters for binary mixtures of surfactant–hydrolysate, as well as for the individual components of the mixtures in the air/water system.

	Adsorption Parameters	−∆G_ads_ [kJ/mol]	Γ_∞_ [μmol/m^2^]	A_min_ [nm^2^]	γ = 60 [mN/m]	
Solution					X	β
Single-component system	Glucopone^®^215	26.26	3.42	0.40	-	-
	Glucopone^®^600	48.89	1.29	1.28	-	-
	0.02Ac-H	22.76	1.17	1.42	-	-
	0.1Ac-H	15.63	2.58	0.65	-	-
	Ac-FD	33.54	0.41	4.04	-	-
Binary system	Glucopone^®^215: 0.02Ac-H	29.44	2.75	0.60	0.42	−13.12
	Glucopone^®^215: 0.1Ac-H	28.58	2.94	0.57	0.40	−13.26
	Glucopone^®^215: Ac-FD-H	27.22	3.34	0.50	-	-
	Glucopone^®^600: 0.02Ac-H	42.82	1.56	1.06	0.47	−36.90
	Glucopone^®^600: Ac-FD-H	42.16	1.67	0.99	0.42	−34.73

The β-parameter represents intermolecular interaction strength, while X refers to the mole fraction of the hydrolysate at the air/water interface.

**Table 4:** Adsorption parameters for modified starch hydrolysates in the toluene–water system.

Parameter	Unit	0.02Ac-H	0.1Ac-H	Ac-FD-H	Ac-Ox-H
Γ_∞_	μmol/m^2^	0.32	0.59	0.20	0.24
A_min_	nm^2^	5.14	2.82	8.14	6.68
−∆G_ads_	kJ/mol	18.31	14.50	26.11	18.81

## Data Availability

The original contributions presented in the study are included in the article, further inquiries can be directed to the corresponding author.

## Abbreviations

Ac-FD-H	hydrolysate of cross-linked acetylated starch phosphate
Ac-Ox-H	hydrolysate of oxidized acetylated starch with an oxidation degree of 0.04 and an acetylation degree of 0.1
0.02Ac-H	hydrolysate of acetylated starch with a degree of substitution of 0.02
0.1Ac-H	hydrolysate of acetylated starch with a degree of substitution of 0.1
Akofine	Akofine P™—fully saturated, deodorized vegetable oil
Amin	area occupied by a single statistical molecule in the monomolecular layer at the saturated phase boundary
Asz and Bsz	the Szyszkowski coefficients
CMC	critical micelle concentration
CRMR	continuous recycled membrane reactor
CTAB	hexadecyltrimethylammonium bromide
C^0^_1,t_ and C^0^_2,t_	total molar concentrations of individual surfactant components 1 and 2
*D*	diffusion coefficient
*DS*	degree of substitution
MAG	1-(7Z-tetradecenoyl)-rac-glycerol
SDS	sodium dodecyl sulfate
Triton™ X-100	polyethylene glycol tert-octylphenyl ether
β	intermolecular interaction strength parameter
γ	surface/interfacal tension
Γ_∞_	saturated phase boundary
γ_CMC_	surface tension in the CMC point
ΔG_ads_	free energy of adsorption
γ_CMC_	surface tension in CMC point
Xi	molar surface fractions in the mixed adsorption layer
